# Age-related characteristics in differentiated thyroid cancer: a 20-year single-center retrospective analysis in pediatric and adolescent patients

**DOI:** 10.20945/2359-4292-2024-0333

**Published:** 2025-04-03

**Authors:** Laura Leite-Almeida, Rita Santos Silva, Margarida Vicente-Ferreira, Carla Costa, Norberto Estevinho, Cíntia Castro-Correia, Sofia Ferreira, Maria Bom-Sucesso

**Affiliations:** 1 Pediatrics Department, Unidade Local de Saúde São João, Porto, Portugal; 2 Faculty of Medicine, Universidade do Porto, Porto, Portugal; 3 Pediatric Surgery Department, Unidade Local de Saúde São João, Porto, Portugal; 4 Pediatric Endocrinology Unit, Pediatrics Department, Unidade Local de Saúde São João, Porto, Portugal; 5 Pediatric Oncology Department, Unidade Local de Saúde São João, Porto, Portugal; 6 PaedCan, European Reference Network for Paediatric Cancer, Vienna, Austria

**Keywords:** Adolescents, Follicular thyroid cancer, Thyroid cancer, papillary, Pediatrics, Thyroid neoplasms

## Abstract

**Objective:**

To characterize a cohort of pediatric thyroid cancer patients, focusing on clinical features and outcomes stratified by age. Subjects and

**methods:**

This retrospective analysis included 63 pediatric differentiated thyroid cancer patients treated at a Portuguese pediatric reference hospital over a period of 20 years. Data extracted from clinical records covered demographics, clinical presentation, family history, tumor characteristics, treatment modalities, complications, disease status, and survival outcomes. Patients younger than 12 years were compared to those aged 12 and older.

**Results:**

The mean age at diagnosis of the sample was 14.5 years, with a preponderance of female patients. Clinical presentation varied significantly between age groups; younger patients were more likely to present palpable cervical lymph nodes, while older patients frequently had solitary thyroid nodules. Family history and identifiable risk factors were similar across groups. However, older patients had higher rates of prior neoplasia and radiation exposure. Age also influenced surgical treatment and outcomes, including complications and recurrence rates.

**Conclusion:**

Our findings corroborate previous evidence on the predominance of papillary carcinoma and the association between radiation exposure and thyroid cancer. Younger patients demonstrated more aggressive tumor characteristics and higher recurrence rates, underscoring the need for age-specific management strategies. Early detection, comprehensive surgical intervention, and multidisciplinary follow-up are essential for achieving optimal outcomes.

## INTRODUCTION

Thyroid cancer represents the most prevalent endocrine malignancy (^1-3^), with primary malignant thyroid tumors being classified into differentiated thyroid cancer (DTC), including papillary or follicular types, medullary thyroid cancer, and less common forms such as anaplastic carcinoma and lymphoma. Notably, papillary thyroid cancer constitutes over 85% of pediatric thyroid cancer cases (^3-10^).

Differentiated thyroid cancer in children is increasingly recognized on a global scale (^1,3,10-12^), attributed to advancements in detection and registration techniques alongside an increase in incidence. Moreover, thyroid cancer has a higher prevalence in girls than in boys, and its incidence increases with age (^1,11,12^). While the majority of pediatric cases are sporadic, some patients have specific risk factors, such as prior exposure to ionizing radiation, genetic predispositions, or congenital goiter resulting from dyshormonogenesis (^1,10-12^).

Typically, DTC manifests as solitary nodules, coinciding with normal thyroid function and absent additional symptoms (^2,7,8,13^). The childhood thyroid gland shows particular vulnerability to radiation and subsequent carcinogenesis, thus rendering pediatric thyroid cancer cases more aggressive with an elevated risk of metastasis and recurrence (^8^). Despite this, the overall prognosis remains generally favorable (^6,7^), with mortality rates among patients under 20 years old being less than 0.1 per 10 million person-years (^11^).

Treatment protocols for thyroid cancer often mandate aggressive surgical intervention (i.e., total thyroidectomy with lymphadenectomy) and may include postoperative radioactive iodine (I-131) therapy (^3,7,10^). The introduction of I-131 has substantially enhanced the prognosis of thyroid cancer, with a strong consensus regarding its benefits for patients at intermediate to high risk. Contemporary guidelines advocate for the individualized dosing of I-131, taking into account the patient’s risk level and body weight or surface area, thereby aiming to optimize efficacy while minimizing potential long-term adverse effects. Excessive use of I-131 is associated with an increased risk of secondary malignancies over time (^10,12,14^). In addition, the long-term follow-up data on pediatric patients with thyroid neoplasms remains limited.

Given this context, our study aimed to characterize a cohort of pediatric thyroid cancer patients, focusing on clinical features and outcomes stratified by age. We delineated the demographic and clinical profiles of patients under 18 years of age diagnosed with thyroid cancer and treated at our center over the last 20 years. Additionally, this research sought to compare the clinical characteristics and outcomes between patients younger than 12 years and those aged 12 and above.

## METHODS

This retrospective analysis included 63 pediatric patients (aged zero to 18 years) with DTC treated over the past 20 years at *Unidade Local de Saúde de São João*, the reference hospital for pediatric oncology in Northern Portugal. Clinical records were reviewed to collect data on age at diagnosis, sex, clinical presentation, family history or risk factors for thyroid cancer, histological type, tumor size, lymph node involvement, treatment, complications, recurrent or persistent disease, distant metastases, levothyroxine supplementation, non-thyroid second neoplasms, and survival. Histological type, lymph node involvement, and tumor size - defined as the carcinoma’s largest dimension - were assessed through a pathological review. Surgical data were extracted from surgical records.

Comparisons were made between patients younger than 12 years and those older than 12. The choice of 12 years as a cutoff was based on the general absence of specific puberty data and the perception that this age often marks the average onset of puberty, which may influence tumor behavior. Although age is an imprecise marker for pubertal status, this cutoff enables easier comparisons with previous studies and is consistent with the common practice in the literature (^4,8^).

Patients at our center underwent surgery (total thyroidectomy or lobectomy, followed by total thyroidectomy if malignancy was confirmed pathologically). Neck dissection was undertaken when cervical lymph node metastases were confirmed pathologically or were macroscopically evident during surgery, either initially or in subsequent surgeries. I-131 treatment was recommended for patients with intermediate and high risk. Follow-up assessments included periodic neck ultrasound, thyroglobulin (Tg), antibodies, and thyroid-stimulating hormone evaluations. Other assessments, such as whole-body I-131 scans and thoracic computed tomography, were considered on a case-by-case basis for patients with detectable but non-rising Tg levels and no identifiable focus on neck ultrasound (^10^).

Postoperative disease risk was stratified according to American Thyroid Association (ATA) Adult Guidelines (^15^) into low risk (papillary tumor with ≤ 5 microscopic metastases [< 0.2 cm] to central compartment neck lymph nodes and serum non-stimulated Tg < 1 ng/mL postoperatively); intermediate risk (papillary tumor with substantial central compartment [1a] lymph node metastases [n = > 5 or > 0.2 cm] or minimal lateral neck [1b] lymph node metastases [n = ≤ 10 and ≤ 3 cm]); and high risk (papillary tumor with extensive lateral compartment [1b] metastases [n = > 10 or > 3 cm] or locally invasive tumor into skeletal muscle, larynx, trachea, esophagus, blood vessels, or nerves, with or without metastases).

Disease status was classified as “recurrent” if a patient showed no evidence of tumor by radioactive iodine imaging and/or neck ultrasound and had undetectable Tg in the absence of interfering antibodies within 1 year after the first surgery but was later found to have evidence of disease upon reoperation; and “persistent” if a patient presented with evidence of tumor by radioactive iodine imaging and/or neck ultrasound, unstimulated Tg > 0.2 ng/mL or stimulated Tg > 1 ng/mL from diagnosis (^12^).

Statistical analysis was conducted using Statistical Package for Social Sciences (SPSS), version 24 (IBM Corp., Armonk, NY). Categorical variables are reported as counts (proportions), and continuous variables as mean (standard deviation [SD]) or median (interquartile range), as appropriate. Continuous variables were compared using the Student’s t-test or the Mann-Whitney U test, and categorical variables with Pearson’s Chi-squared test; p-value < 0.05 was considered statistically significant.

All stages of the study adhered to the Ethical Principles for Medical Research Involving Human Subjects of the Declaration of Helsinki. The study was approved by the ethics committee of the Faculty of Medicine at the *Universidade do Porto* (protocol no. 133/18).

## RESULTS

Over the past 20 years, our center has treated 63 pediatric patients (aged zero to 18 years) with DTC. The mean age at diagnosis was 14.50 years (SD = 3.80), with females comprising 77% (n = 49) of the cohort.

Regarding the age at diagnosis, 13 patients were younger than 12 (mean age 8.70 years, 61.5% female; one had a neonatal diagnosis), while 50 patients were older than 12 (mean age 16.00 years, 82.00% female). Table 1 presents the clinical characteristics of the patients according to their age group.

**Table 1:** Clinical characteristics of the patients (n = 63) according to their age group

Variables		< 12 years old (n = 13)	≥ 12 years old (n = 50)	p-value
Age at diagnosis, years		8.7 ± 3.50	8.7 ± 3.50	0.012^*^
Female		8 (61.50)	8 (61.50)	0.114^†^
Clinical presentation				0.036^†^
	Solitary thyroid nodule	2 (18.20)	28 (58.30)	
	Palpable cervical lymph node	5 (45.50)	5 (10.40)	
	Asymptomatic screening with thyroid ultrasound	2 (18.20)	11 (22.90)	
	Dysphonia	1 (9.10)	2 (4.20)	
	Enlarged thyroid	1 (9.10)	2 (4.20)	
Family history of thyroid disease				0.433^†^
	None	8 (66.70)	38 (77.60)	
	Benign	3 (25.00)	8 (16.30)	
	Malign	1 (8.30)	3 (6.10)	
Known risk factor for thyroid neoplasm at admission				0.778^†^
	None	12 (92.30)	37 (75.50)	
	Previous ionizing radiation	0	5 (10.20)	
	Hodgkin lymphoma	0	3	
	Ependymoma	0	2	
	Previous neoplasia without radiotherapy	1 (7.70)	4 (8.20)	
	Ependymoma	1	0	
	Burkitt lymphoma	0	1	
	Adrenal neuroblastoma	0	1	
	Uterine rhabdomyosarcoma	0	1	
	Wilms tumor	0	1	
	Hashimoto thyroiditis	0	1 (2.00)	
	Congenital goiter	0	1 (2.00)	
	Smoking	0	1 (2.00)	
Initial TSH concentration, mIU/L		2.4 (1.2-3.8)	1.8 (0.9-2.7)	0.344^‡^
Histological type				0.970^†^
	Papillary thyroid cancer	11 (91.70)	46 (92.00)	
	Follicular thyroid cancer	1 (8.30)	4 (8.00)	
Tumor size in cm		1.8 ± 1.2)	1.9 ± 1.3)	0.195^*^
Lesion number and location				0.308^†^
	Unifocal	8 (72.70)	24 (55.80)	
	Multifocal	3 (27.30)	19 (44.20)	
Extrathyroidal extension		8 (66.70)	9 (21.40)	0.003^†^
Cervical lymph node metastasis				0.022^†^
	None	4 (33.30)	34 (75.60)	
	Central compartment	2 (16.70)	3 (6.70)	
	Central and lateral compartments	6 (50.00)	8 (17.80)	
Distant metastasis	0	3 (7.00)	0.347^†^

Results expressed as mean ± standard deviation or n (%) or median (interquartile range).

* Student’s t-test; †Pearson’s Chi-squared test; ‡Mann-Whitney U test.

TSH: thyroid-stimulating hormone.

The clinical presentation varied significantly between both groups. Solitary thyroid nodules were more common in older patients (58.30% versus 22.90%), whereas palpable cervical lymph nodes were more prevalent among younger patients (45.40% versus 10.40%). Dysphonia and enlarged thyroid were present in both groups but occurred more frequently in older patients. Approximately one-fifth of the patients in each group were asymptomatic.

The family history did not differ significantly between groups, and most patients had no identifiable risk factors. Among the older patients, nine had a history of previous neoplasia (five with prior radiotherapy exposure and four without). Conditions such as Hashimoto’s thyroiditis, congenital goiter, and smoking were only reported in older patients.

Tumor characteristics, including type, size, number, and location, did not significantly differ between groups. However, extrathyroidal extension and cervical lymph node metastasis were more prevalent in patients diagnosed before the age of 12.

The initial treatments and outcomes for both groups are presented in Table 2. The options for surgery were similar between groups, with total thyroidectomy being the preferred choice. Neck dissection, either central and/or lateral, was performed in half of the younger patients and 29.5% of the older ones. Surgical complications, including recurrent laryngeal nerve injury and transient hypoparathyroidism, were more commonly observed in the younger group.

**Table 2:** The treatments and outcomes of the patients (n = 63)

Treatments and outcomes		< 12 years old (n = 13)	≥ 12 years old (n = 50)	p-value
Surgery				
	Total thyroidectomy	9 (75.00)	33 (66.00)	0.136^‡^
	Initial lobectomy + TT in a second surgery	3 (25.00)	15 (30.00)	
	Lobectomy	0 (0.00)	2 (4.00)	
Neck dissection (central and/or lateral)		6 (50.00)	13 (29.50)	0.185^‡^
Surgical complications				
	Recurrent laryngeal nerve injury	3 (25.00)	2 (4.10)	0.001^‡^
	Transitory hypoparathyroidism	2 (16.70)	0	
	Permanent hypoparathyroidism	0	5 (10.20)	
Risk for persistent postoperative disease^*^				
	Low	6 (50.00)	30 (63.80)	0.071^‡^
	Intermediate	3 (25.00)	2 (4.30)	
	High	3 (25.00)	15 (31.90)	
Postoperative radioactive iodine therapy				
	Number of patients	13 (100.0)	38 (76.00)	0.050^‡^
	Number of treatments	1 (^1-3^)	1 (^1-1^)	0.139§
	Number of treatments	2.1 ± 1.4	1.5 ± 1.3	0.196^¶^
	Dose, mCI	100.0 (52.5-143.3)	100.0 (77.5-120.8)	0.987^§^
Extrathyroidal extension				
	No disease	7 (53.80)	36 (72.00)	0.210^‡^
	Persistence	4 (30.80)	13 (26.00)	0.730^‡^
	Recurrence	2 (15.40)	1 (2.00)	0.044^‡^
	Mortality	0	2 (4.00)	0.664^‡^

Results expressed as n (%) or median (interquartile range) or mean ± standard deviation.

* Classified as low-, intermediate-, and high-risk tumors according to the American Thyroid Association (^14^);

† Student’s t-test,

‡ Pearson’s Chi-squared test;

§ Mann-Whitney U test.

TT: total thyroidectomy.

The rates of persistent postoperative disease were similar in both groups, although recurrence was higher in younger patients. There were two deaths, both in patients who had secondary thyroid neoplasia, primarily associated with complications from previous malignancies and their treatments.

## DISCUSSION

Thyroid cancer was predominately found in females, with papillary carcinoma being the predominant histopathologic diagnosis, which is consistent with prior research (^3-12^). Children under 12 years typically presented with palpable cervical lymph nodes, while older patients more often exhibited solitary thyroid nodules. This pattern aligns with existing literature, which identifies palpable neck masses as a common initial presentation (^7,13^).

Across all age groups, family history and rates of previous neoplasia remained similar. However, older patients reported greater exposure to ionizing radiation. Researchers have indicated the existence of a dose-response relationship between ionizing radiation exposure and the risk of developing thyroid cancer in children; the risk increases to a plateau at 10 to 30 Gy, then declines, likely a result of cell-killing effects at higher doses (^1,10,12,16,17^). Thus, monitoring for thyroid cancer is pivotal in childhood cancer survivors who have undergone radiotherapy, particularly within the dose range linked to heightened risk. Among our cohort, we observed five patients with secondary thyroid neoplasia, all of whom had previously received radiotherapy for primary tumors and were diagnosed during surveillance.

Tragically, two patients succumbed to their illnesses. Notably, both deaths were primarily due to complications from their previous malignancies and radiation therapies, not directly from the thyroid cancer itself. One boy, initially diagnosed at age 9 with grade III ependymoma of the posterior fossa and later treated with radiotherapy, was subsequently diagnosed with thyroid cancer presenting lung metastasis and, at 15, with metastatic glioblastoma, which ultimately led to his death. Another boy, diagnosed with Hodgkin disease at 4 years old and treated with radiotherapy, developed highly invasive thyroid cancer with distant metastasis 10 years later and died at 24 years old from heart failure with pulmonary hypertension. These cases underscore the importance of long-term monitoring for individuals exposed to radiation during childhood (^10,16,17^).

Extrathyroidal extension and cervical lymph node metastasis were more frequently observed in younger patients. Distant metastasis, predominantly involving the lung, was exclusive to older patients, affecting 4.80% of the total cohort. This incidence is lower than what has been previously reported in the literature (^6,10^), possibly due to the smaller number of younger patients in our study (n =13) since earlier research suggested greater aggressiveness of thyroid cancer in this demographic, with increased extrathyroidal extension, and higher rates of lymph node and lung involvement (^6,7,10,12^). The question of whether younger age independently contributes to a higher risk of extensive disease or recurrence remains, as treatment strategies, genetic predisposition, and radiation exposure may also influence outcomes. Moreover, the potential impact of pubertal development on DTC behavior warrants consideration. The ATA recommends incorporating pubertal assessments, such as the Tanner and Marshall criteria, into future research (^12^). Our study utilized age as a surrogate for pubertal status, a method that is not without limitations.

Recurrence rates were notably higher in younger patients, particularly concerning local or cervical lymph node recurrences, while rates of persistent disease were comparable across age groups. Younger patients experienced more surgical complications and were more frequently treated with I-131 therapy. Recurrent laryngeal nerve injury was the most common surgical complication, corroborating other findings (^2,10^). Permanent hypoparathyroidism was identified in five patients, one of whom had the parathyroids included in the biopsy sample. The heightened occurrence of surgical complications in younger patients aligns with ATA guidelines, highlighting an increased risk for patients under 10 years of age, especially in cases involving extrathyroidal extension, lymph node dissection, and subsequent surgeries (^12^). This elevated risk is likely due to the significantly smaller anatomical space in younger children, complicating the isolation and preservation of the recurrent laryngeal nerve and parathyroids (^18^).

Unlike other studies reporting multifocality rates of 51 to 66% (^6,19^), most of our patients exhibited unifocal lesions. This discrepancy can be attributed to earlier diagnosis, facilitated by Portugal’s robust primary healthcare system and comprehensive infant health program. These programs ensure regular medical surveillance and easy access to acute care appointments. Upon suspicion of DTC, prompt referrals to oncology specialists at one of the four central oncology centers, like ours, are straightforward. Urgent appointments and the initiation of diagnostic investigations typically occur within a week. Furthermore, cancer survivors receive long-term, close follow-up, aiding early detection in this small, yet significant, group.

We highlight the extraordinarily rare case of neonatal thyroid cancer, previously published (^20^). The subject is a full-term female neonate who presented with respiratory distress due to airway compression by a large cervical tumor, histologically confirmed as follicular thyroid carcinoma. At the age of 7 days, she underwent near-total thyroidectomy, resulting in transient tracheomalacia and unilateral vocal cord paralysis, and received I-131 therapy at the age of five months.

The strength of our study resides in its substantial pediatric patient cohort, providing valuable data on the presentation, management, and outcomes of thyroid cancer. This is particularly significant considering the disease’s rarity and the relative scarcity of studies concentrating on specific pediatric age groups. Nevertheless, the study is limited by its retrospective nature, relying on existing medical records for data collection. This proved particularly challenging when assessing the cause of persistent disease. Additionally, the past 20 years have seen significant changes in clinical practices and approaches to these patients. The ATA guidelines for children were first published in 2015, followed by European Thyroid Association Guidelines in 2022 (^10,12^). Prior to these publications, treatment approaches were less standardized, leading to broader use of radioiodine therapy, even among patients considered low-risk by current standards. This accounts for the application of radioiodine therapy in patients over 12 years of age with low-risk profiles, as well as its use in younger patients. Moreover, the younger patients comprised only 13 individuals, potentially affecting our comparisons.

Multidisciplinary follow-up at specialized centers is critical (^10^), involving pediatric endocrinologists, oncologists, surgeons, radiologists, and psychologists to support both the patient and their family. The authors emphasize the importance of adhering to international guidelines, such as those published by the ATA and European Thyroid Association (ETA), to standardize diagnosis, treatment, and follow-up, thereby optimizing the prognosis of this condition (^10,14^). Hence, establishing international databases is imperative for standardizing care, especially considering the rising incidence of thyroid carcinoma in children.

In summary, pediatric thyroid cancer presents differently across age groups, with younger patients exhibiting more aggressive features and higher recurrence rates. These findings underscore the need for age-specific management strategies to optimize outcomes. Each case of pediatric thyroid cancer necessitates a tailored approach, considering the disease’s unique aspects in children. Early detection, appropriate surgical intervention, and continuous monitoring are crucial for favorable outcomes, emphasizing the importance of a multidisciplinary team in a reference center.
